# Retrospective study maxillofacial fractures epidemiology and treatment plans in Southeast of Iran

**DOI:** 10.4317/medoral.20652

**Published:** 2015-06-27

**Authors:** Sahand Samieirad, Elahe Tohidi, Akbar Shahidi-Payam, Maryam-Alsadat Hashemipour, Ali Abedini

**Affiliations:** 1 DDS, MSc. Assistant Professor of Oral and maxillofacial surgery, Department of Oral and Maxillofacial Surgery, School of Dentistry, Mashhad University of Medical Sciences, Mashhad, Iran; 2 DDS, MSc. Assistant Professor of Oral & Maxillofacial Radiology, Department of Oral & Maxillofacial Radiology, School of Dentistry, Mashhad University of Medical Sciences, Mashhad, Iran; 3 DDS, MSc. Member of Kerman Social Determinants on Oral Health Research Center, Kerman University of Medical Sciences, Kerman, Iran. Assistant Professor of Oral and maxillofacial surgery, Department of Oral and Maxillofacial Surgery, School of Dentistry, Kerman University of Medical Sciences, Kerman, Iran; 4 DDS, MSc. Member of Kerman Social Determinants on Oral Health Research Center, Kerman University of Medical Sciences, Kerman, Iran and Kerman Dental and Oral Diseases Research Center , Kerman University of Medical Sciences, Kerman, Iran. Associate Professor of Oral Medicine, Department of Oral Medicine, School of Dentistry, Kerman University of Medical Sciences, Kerman, Iran; 5 DDS. Dentist. Kerman Dental and Oral Diseases Research Center. Kerman University of Medical Sciences, Kerman, Iran

## Abstract

**Background:**

The epidemiology of facial injuries varies in different countries and geographic zones. Population concentration, lifestyle, cultural background, and socioeconomic status can affect the prevalence of maxillofacial injuries. Therefore, in this study, we evaluated the maxillofacial fractures epidemiology and treatment plans in hospitalized patients (2012-2014) which would be useful for better policy making strategies.

**Material and Methods:**

In this retrospective study, the medical records of 386 hospitalized patients were evaluated from the department of maxillofacial surgery at Bahonar Hospital of Kerman, Iran. The type and cause of fractures and treatment plans were recorded in a checklist. For data analysis, ANOVA, t-test, Chi-square, and Fisher’s exact test were performed, using SPSS version 21.

**Results:**

The majority of patients were male (76.5%). Most subjects were within the age range of 20-30 years. Fractures were mostly caused by accidents, particularly motorcycle accidents (MCAs), and the most common site of involvement was the mandible (parasymphysis). There was a significant association between the type of treatment and age. In fact, the age group of 16-59 years under went open reduction internal fixation (ORIF) more than other age groups (*P*=0.02). Also, a significant association was observed between gender and the occurrence of fractures (*P*=0.01).

**Conclusions:**

Considering the geographic and cultural indices of the evaluated population, it can be concluded that patients age and gender and trauma causes significantly affect the prevalence of maxillofacial traumas and fracture kinds and treatment plans.

**Key words:**Epidemiology, treatment, facial injuries, face fractures, maxillofacial trauma, trauma.

## Introduction

Traumas arising from physical injuries are the most common type of trauma and may occur due to various reasons. Considering the prevalence of physical traumas and their deleterious effects on individuals, these injuries are among the major health concerns, worldwide (1). In fact, in the United States, accidents are the third cause of death in all age groups (2,3).

Disregard for safety while driving, working, and performing daily activities can result in physical traumas. Moreover, treatment and rehabilitation are associated with psychological problems, severe morbidities, disabilities, and mental damages. In addition, these traumas impose a significant financial burden on individuals and societies (4,5).

Facial soft and hard tissue injuries may be caused by occupational injuries, falls, motor vehicle accidents (MVAs), sports injuries, and interpersonal violence (6). The epidemiology of facial injuries varies in different countries and cities and geographic zones. Population concentration, lifestyle, cultural background, and socioeconomic status can affect the prevalence of maxillofacial injuries (7).

Several studies have investigated the epidemiology of facial injuries in different countries and populations (8-13). However, there is still limited data regarding the epidemiology and treatment of facial injuries in developing countries, especially in Iran. Some researchers have studied the prevalence of maxillofacial fractures in different provinces and regions of Iran (14-16). However, there is still insufficient information available about the etiology and outcomes of these injuries especially in Kerman province.

In Iran, MVAs are the most common cause of maxillofacial fractures, and the rate of these accidents is following a rising trend (17,18). Maxillofacial fractures are classified as serious injuries, given the specific anatomic features of jaw and face; these injuries are also more common among men and 20-30-year-old individuals (19).

 Facial fractures are of grave importance, considering the adverse socioeconomic and psychological consequences for patients. Therefore, in this study, with the aim to expand the available statistical data in Iran, we evaluated the incidence of maxillofacial fractures in hospitalized patients, based on age and gender and type and treatment plan in the Oral and Maxillofacial Surgery Department of Bahonar Hospital of Kerman, located in south east of Iran during 2012-2014.

## Material and Methods

- Patients 

In this retrospective cross-sectional study, census sampling was applied. All patients were completed Informed consent form before including in the study. Patients, admitted to the Oral and Maxillofacial Surgery Department at Bahonar Hospital of Kerman during 2012-2014, were included in the study. The sample size was calculated at 386 subjects. The exclusion criteria were as follows: 1) the immediate treatment of outpatients without hospitalization; 2) patients with only dentoalveolar fractures which were redacted by arch bar without hospitalization; 3)non-completed or incomplete medical records; 4)undergoing other procedures such as opening of the arch bar or removal of a plate in patients whom underwent maxillofacial surgeries before; 5)patients with only soft tissue injuries who were treated in emergency room without hospitalization; and 6) unavailability of their cords of patients referring to the otolaryngology department (4,1,20). After excluding these cases, only 221 patients were remained to analysis.

- Methods

All demographic data (e.g., patients age and gender) were collected, and the patients’ medical records were reviewed to extract information related to the date of referral, cause of trauma, patients’ complaints, involved injured bones, concomitant fractures and injuries of soft tissues and other organs, the exact mandibular status, facial examinations, and radiographic images. Data collection tools included observation and census sampling of medical records and documents. Also, we used the archived oral and maxillofacial radiology reports at the surgery department of the hospital.

Maxillofacial fractures were treated using the following methods in our department: 1) closed reduction (CR); 2) open surgical treatment or open reduction & internal fixation (ORIF); 3) follow-up and reevaluation of the status of suspected fractures (without any specific treatments).

- Ethical considerations

Ethical considerations were taken into account throughout the study, and the patients’ names and medical information remained completely confidential. The subjects’ medical history was used solely for the purposes of the current study. The research proposal was approved by the ethics committee of Kerman University of Medical Sciences with the 289.93.k code.

- Statistical analysis 

We used descriptive statistics such as distribution and continuity (mean and standard deviation) for representing the obtained data. For data analysis, t-test was performed to compare the variables between females and males. ANOVA test was used for the comparison of variables in more than two groups, based on the cause, location, and year of the accident. Moreover; Chi-square was performed to assess the association between qualitative and quantitative variables. In this study, the significance.

Level was considered at 0.05, and SPSS version 21 was used for statistical analysis.

## Results

Of 221 patients, 169 cases (76.5%) were male and 52 subjects (23.5%) were female. The mean age of subjects was 26.9±12 years (age range: 1-71 years). As it can be seen in  Table 1, the majority ofsubjects were within the age range of 20-30 years. The highest rate of fractures occurred in summer (31.22%). In fact, the highest rates were reported in September and October (13.12% each), followed by April (11.76%). In total, 121 and 100 cases were selected in 2013 and 2014, respectively.

**Table 1:**
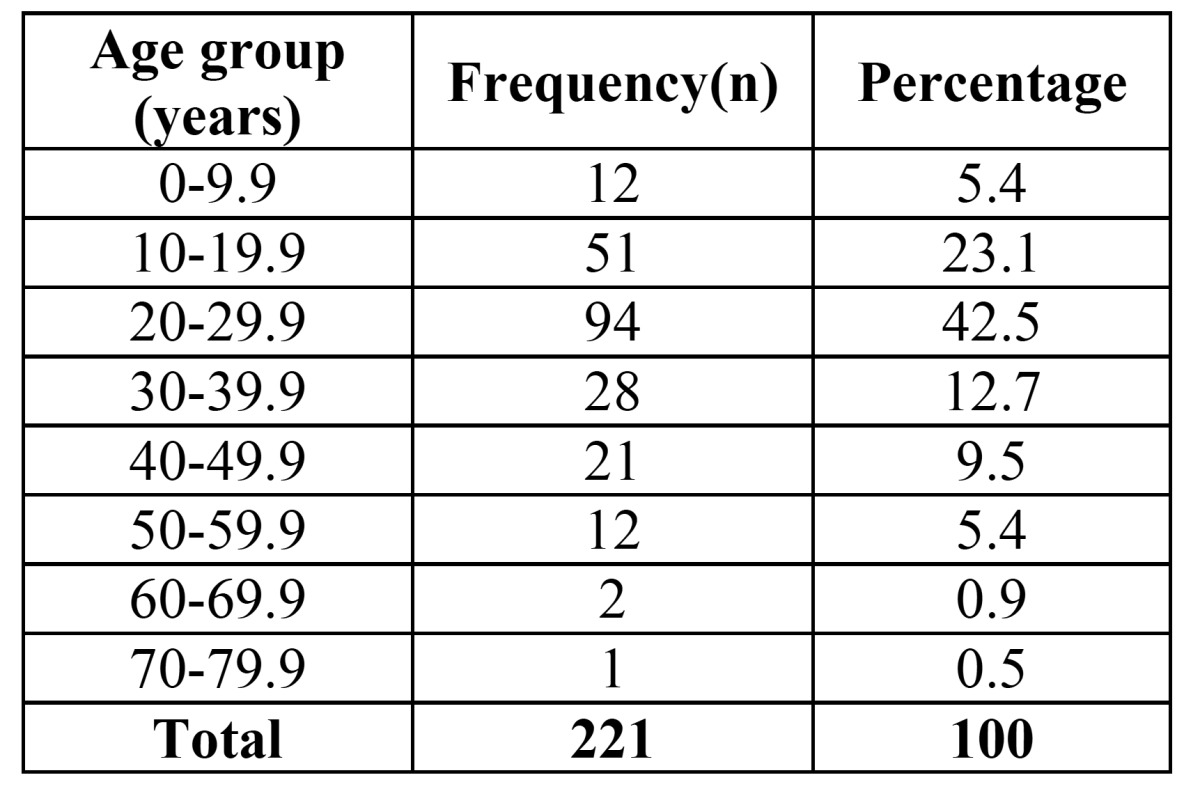
Frequency of maxillofacial fractures in different age groups

MCAs accounted for the majority of traumas (57.5%) followed by CA (13.6%), and occupational injuries had the minimum prevalence (2.3%). In 221 patients, 384 anatomic and bone fractures were reported, and in total, 488 cases of fracture lines were found. We also determined the anatomical location of maxillofacial fractures. It should be noted that the total percentage of fractures in anatomic locations was higher than 100%, given the possibility of having fractures in several locations. Mandibular fractures had the highest frequency (47.1%), followed by nasal bone (43.9%), Zygomaticomaxillary complex (ZMC) fractures (32.1%) ( Table 2).

**Table 2:**
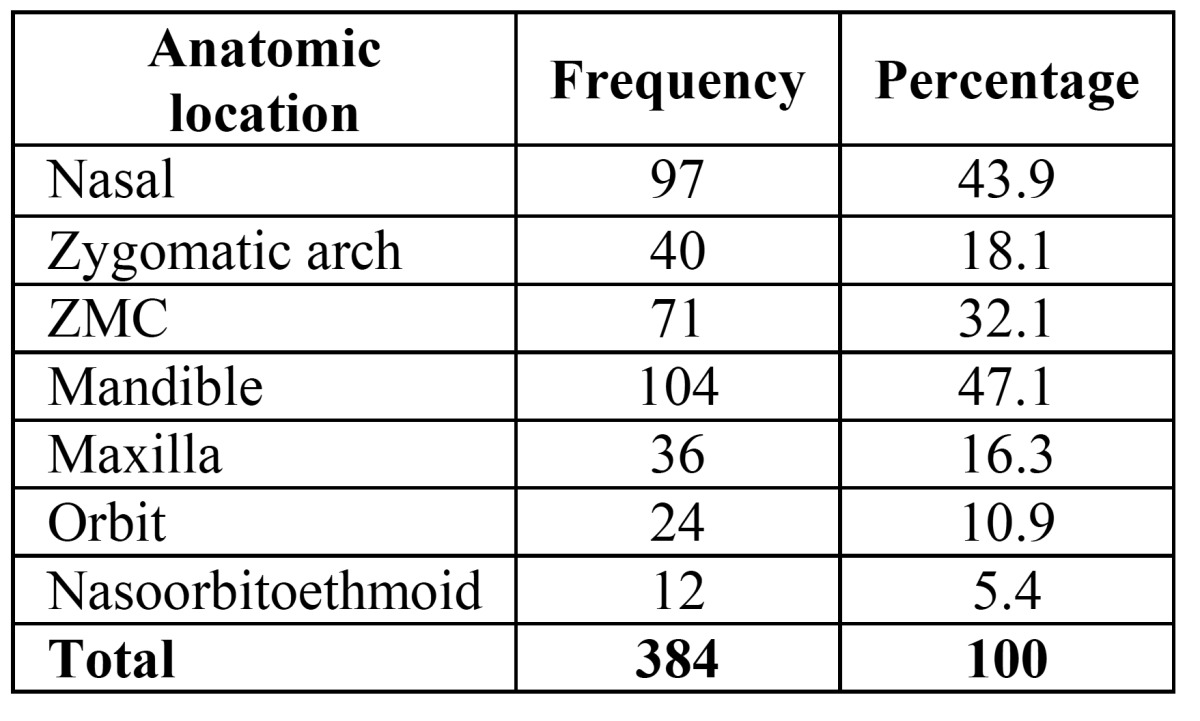
Frequency and percentage of the anatomical location of maxillofacial fractures.

We examined the frequency and distribution of maxillofacial fracture lines in anatomical locations; the findings showed that bilateral fractures were the most common form in the mandible (50%), followed by left-side injuries (27.9%).The most common fracture sides were the left ZMC, zygomatic arch, and the orbit.

The location and anatomical position of fractures were determined in the mandible; 186 lines were detected in 104 fractured mandibles, which was due to variations in mandibular fractures in each patient. The most common anatomical location of mandible fractures was the parasymphysis (25.2%), followed by subcondylar region (18.8%). The lowest number of fractures was recorded in the coronoid area (0.5%) ( Table 3).

**Table 3:**
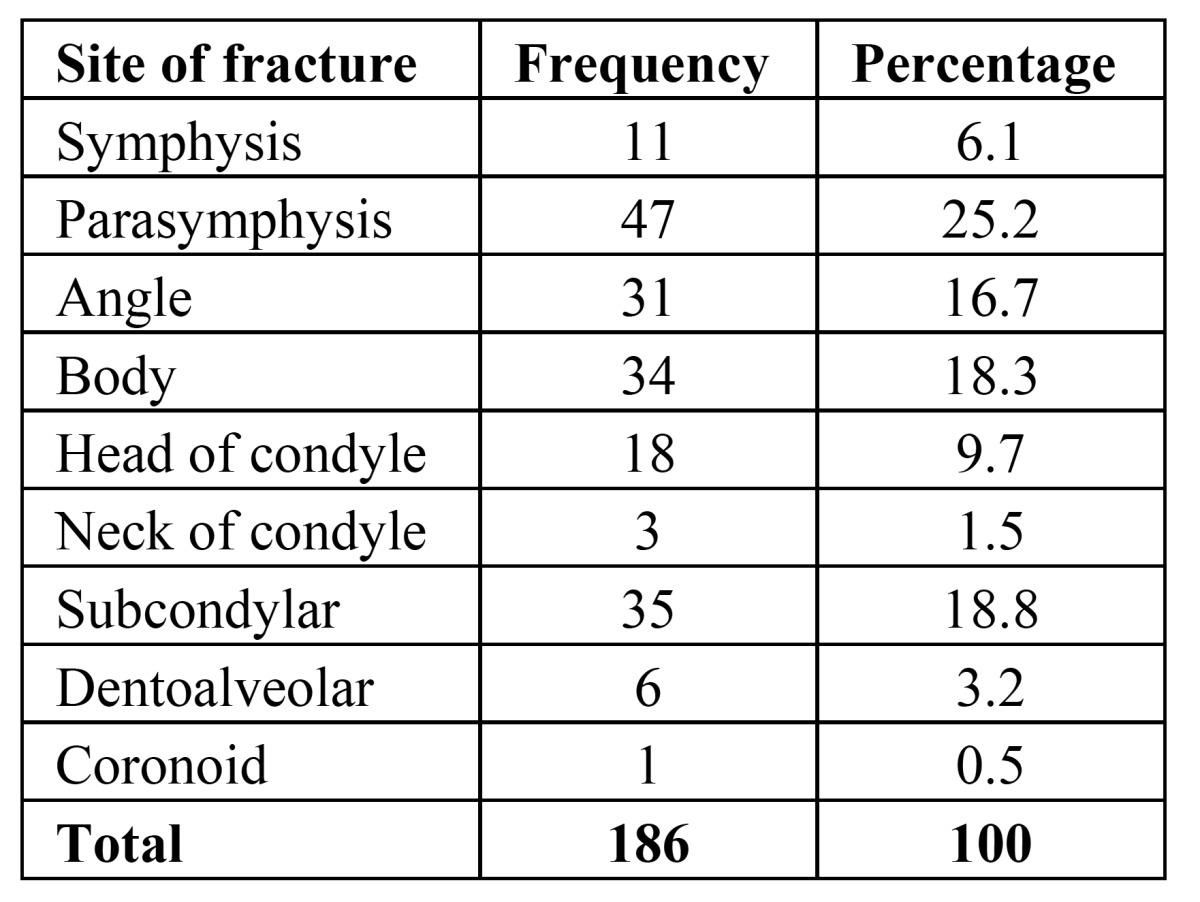
The frequency of the anatomical location of mandibular fractures.

Based on Peterson’s classification (20), if parasymphysis was considered as a portion of symphysis, fracture frequency was estimated at 31.3%. If the head and neck of condyle and subcondylar regions were considered as a single component, the overall incidence of condylar fractures was 30%.

**Figure 1:**
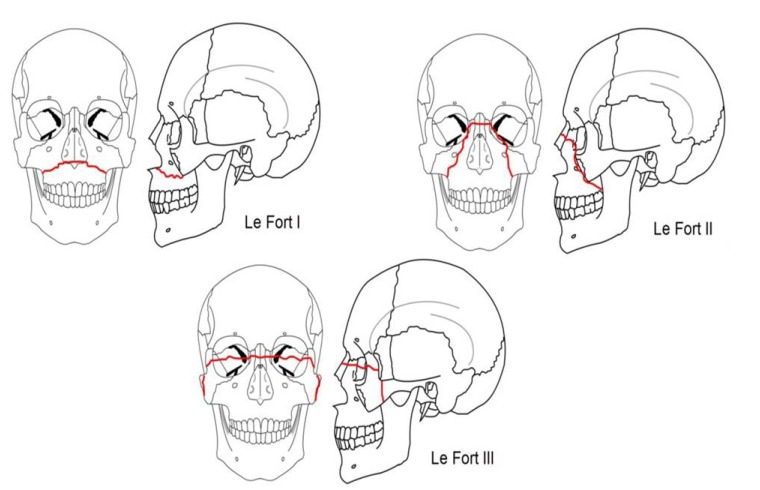
Le Fort classification of maxillary fractures.

A total of 58 fracture lines were reported in 36 patients with fractured maxilla, and the most commonly reported site was Le Fort1 (the palate is separated from the maxilla) with 29% prevalence followed by Le Fort2 (the maxilla separates from the face ) and Le Fort 3(craniofacial dysjunction is present (24.4% and 24%, respectively) (21) (Fig. 1).

Soft tissue traumas were reported in 146 patients (66.1%). Lip injuries were the most prevalent soft tissue traumas, reported in 34.2% of cases, followed by chin, forehead, and eyelid injuries (30.8%, 29.4%, and 21.2%, respectively). Moreover, simultaneous injuries were reported in 97 patients (43.9%). The most common associated injury was orthopedic damage, reported in 47.42% of the patients followed by cranial fractures with 21.64%.

In these cases, the most frequent treatment was CR (64.3%), followed by ORIF (49.3%). It was possible to perform both CR and ORIF for several fractures in one patient simultaneously.

In zygomatic arch fractures, CR accounted for 92.5% of treatments; also, this type of treatment was performed in 92.8% of nasal fractures. However, open treatment was more common in ZMC and orbit fractures (93% and 79.2%, respectively). In condylar fractures of the mandible, CR was performed in 83% of cases. Also, CR was performed in 64.5% of non-condylar fractures including the mandibular symphysis, body, and angular regions.

Considering the categorization of fractures, 108 cases (48.8%) had single fractures and 113 subjects (51.2%) had multiple injuries ( Table 4).

**Table 4:**
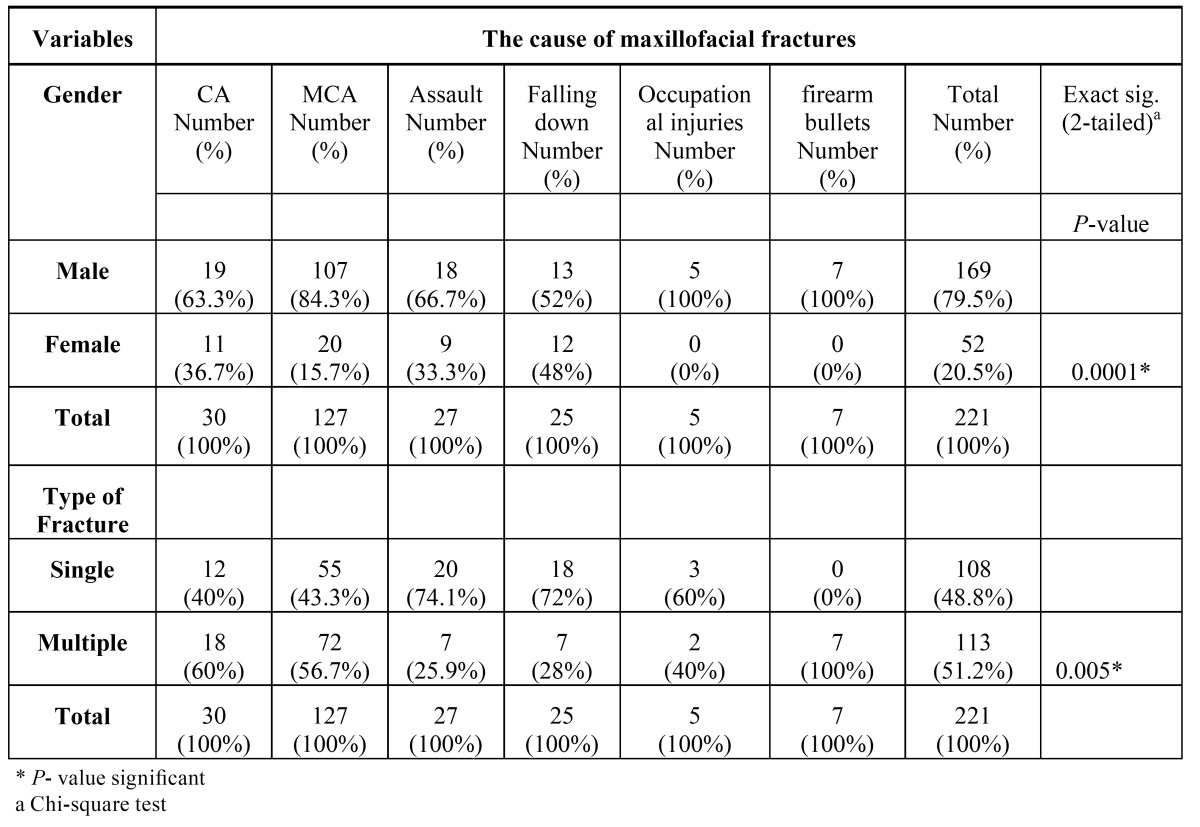
Association between the cause of maxillofacial fractures, gender, and type of fracture.

The prevalence of fractures in males was 3.25 times higher than females (*P*=0.01). The mean age of subjects during the accidents was 26.5±10.7 years in men and 28.2±15.8 years in women; there was no significant difference between years in males and females (*P*=0.6, t- test). A significant association was observed between gender and the cause of fractures, with an exception of falling down cases (*P*=0.03). Males were more prone to MVAs, occupational injuries, and assault, compared to females ( Table 4, Table 5).

**Table 5:**
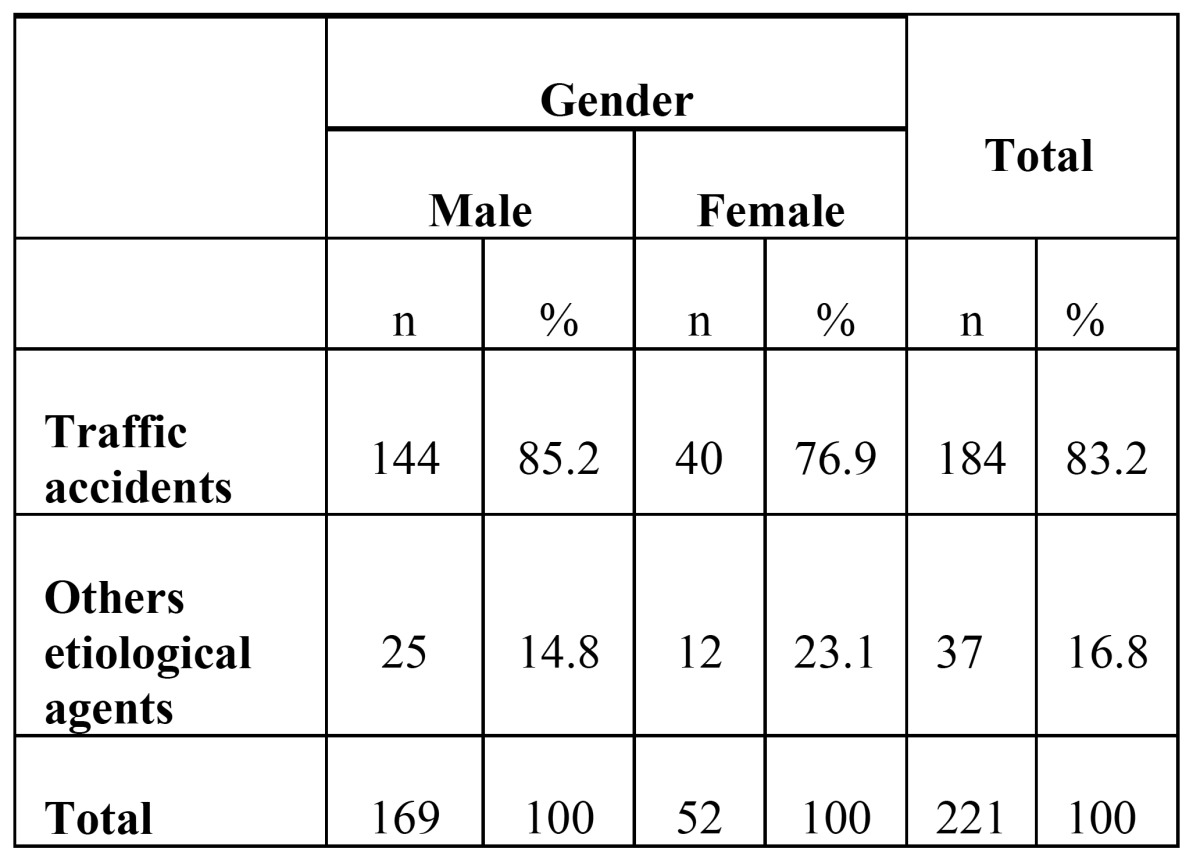
Hospitalization frequency and percentage of traffic accidents and all other etiological agents according to gender.

In cases of assaults, falling downs and occupational injuries, the fracture types were simple and isolated, while in car accidents, especially motorcycle accidents, most fractures were multiple. Incidents involving firearm bullets included 4.5% of all trauma cases. In this regard, Chi-square test showed a significant association between the type of fractures and cause of trauma (*P*=0.005) ( Table 4).

The findings showed that most maxillofacial treatment plan were closed reduction (CR) (64.30%) followed open reduction (29.30%) and no treatment and follow up (6.40%). Also, in the age group of <15 years, most maxillofacial treatment plan were CR (73.7%); CR was also reported in the age group of>60 years (82%). In the age group of 16-59 years, open reduction (ORIF) was the predominant treatment method (54.6%). Fisher‘s exact test showed a significant difference between the type of treatment and age; in fact, the age group of 16-59 years under went ORIF more than other age groups (*P*=0.02).

## Discussion

Kerman is the largest, most developed and most important city in Southeast Iran (population of about 3000000 (22). Also, Oral and Maxillofacial Surgery Department at Bahonar Hospital of Kerman is most equipped, most advanced and most developed Maxillofacial Surgery Department in South-East Iran.

In Kerman, there are some laws against drinking and driving, laws that require the use of seat belts and speed limits for the roads traffic (60 and 80 km/h) (23).

During 3 years, from January 2012 to December 2014, 221 patients were treated by oral and maxillofacial surgery specialists, and the analysis of this sample may provide knowledge about the current distribution of facial fractures in Southeast Iran, as well as help to build a database that may improve medical and dental programs to prevent facial trauma.

Not only maxillofacial traumas can be life-threatening, given the severe bleeding and airway compromising, but facial disfigurement and loss of function are two major consequences of maxillofacial injuries; these injuries may be also followed by blindness or difficulty in jaw function. Therefore, it is of high significance to identify the etiology and epidemiology of maxillofacial traumas. As we showed, the incidence rate of fractures in men was higher than women. This finding was in agreement with several previously conducted studies (1,8,13,14,16,24-30); this shows the alignment of the current research with the mentioned studies.

Bakardjiev and Lee in retrospective studies investigated the prevalence of maxillofacial fractures in southern Bulgaria and Jeju in Korea, respectively (31,32). Correspondingly, they reported a higher number of injuries in males, compared to females.

Male are generally more socially active and more involved in life-threatening activities, sports, and violence.

The highest number of injuries was reported in the age range of 20-30 years, in agreement with other studies (8,13,28,32-35). Iida *et al*. reported that the most frequent age group was the 11-20 groups, because they are more exposed to all the etiological agents assessed than any other age group (27). Due to their wish to enjoy the pleasures of modern life, these subjects are potentially more likely to exceed speed limits, and even get involved in physical conflicts as a result of their increased physical energy.

Also, in this study, the highest number of traumas was recorded in October, which was consistent with the findings by other studies (13,36-38). This result can be explained by this fact that in the summer and autumn in Iran, people tend to be more exposed to risk situations, for example, by engaging more frequently in physical activities, taking part in social reunions, and road trips. All these factors certainly contribute to increase the incidence of major causes of trauma, namely traffic accidents, falls, and aggressions.

The maximum number of fractures was reported in the mandible. There was a significant association between the cause of fractures and gender. In fact, males were more affected by MVAs, violence, and occupational injuries (except falling down) than females. Also, we found a significant association between age and type of treatment.

In our study, the ratio of patient to year was higher than that reported in other investigations such as studies by Mesgarzadeh and Arangio, who assessed the prevalence of maxillofacial fractures in west of Iran and Italy, respectively (39,40).

This study showed that MCAs were the most common cause of fractures (57.5%). This finding was aligned with the results of studies by Van Hout, Momeni, and Mohajerani (17,41,42). However, cultural differences, sports activities, daily activities, and occupational status might affect the etiology of maxillofacial traumas and lead to discrepancies between different studies.

Review of literature showed that the most common cause of facial fractures is associated with traffic accidents (8,27,34,37,43,44), but others have demonstrated that assault is the most frequent etiological agent (44,45). According to the research work by Taher (32), fractures caused by firearm bullets are the most common in Iran. Our results showed a high incidence of fractures caused by traffic accidents, particularly those that involve cars, which is particularly significant among individuals 21-30 years of age. However, this and other etiological agents recorded in this study directly depend on the age and gender of the patient and determine the frequency at which a certain region of the facial skeleton sustains a fracture.

As a result of this research work, it is showed that traffic accidents were the cause of 71% of the cases. Iida *et al*. (27) conducted a retrospective study with 1502 patients with facial fractures and found that traffic accidents accounted for 52% of the cases. The explanation for the high incidence of traffic accidents found in both the study by Iida *et al*. (27) and our study lies in the type of hospital where both studies were carried out, namely, local reference centers for the treatment of trauma.

In the present research work, it is found that assault is the second most frequent etiological agent (12.2%), a finding that is in agreement with other studies (1,37,46). Most patients treated at our hospital in addition to other social and economic problems had a low socioeconomic status. The increase in urban violence observed in Kerman is strongly associated with social/economic conflicts to which many people, especially youngsters, are subjected. Taking into consideration this complex scenario and the current tendency of urban violence and social conflicts to increase, we believe that a potential reduction in interpersonal violence as a major cause of trauma seems to be much more difficult and unlikely than a reduction in facial trauma caused by traffic accidents.

Our findings showed that the mandible was the most involved bone (47.1%) followed by nasal bone fractures(43.9%), results that are in agreement with those reported by other authors (8,13,16,27,34,47,48). Zandi *et al*, Hussain *et al*., showed that nasal bone fractures were the most prevalent type of trauma (3,18,45) which were the second most prevalent maxillofacial fracture in our study. Some other studies (26,49) found that facial fractures in the zygomatic complex were more frequent. Minor differences in the frequency of fractures can be caused by variations in the etiology of fractures in various studies.

Motamedi showed that condylar and parasymphysis regions accounted for the highest number of fractures (14); this finding is in agreement with our obtained results.

The increasing in use of motorbikes has led to a greater number of accidents and, consequently, facial fractures (28). According to Huelke and Compton (43), although car accidents are more frequent, motorbike accidents are usually more serious. Despite the speed limits enforced and respected in, for example, Thailand, accidents result from the difficulty in accepting to wear helmets because of the hot weather (50) High speeds, together with the disrespect for traffic laws, and a frequent disregard for the need to wear a helmet are two problems in Kerman, either due to hot weather or discomfort, which leads to serious, often fatal accidents. According to Subhashraj *et al*. (28), motorbike accidents are more frequent in India due to socioeconomic conditions, speeding, disrespect for traffic laws, poor road conservation, and not wearing a helmet or safety equipment.

From all of injures, facial fractures causes by firearm bullet wounds was determined about 4.5% that is similar to Paes *et al*. (1). Taher (32) reported that 69.04% of the cases were caused by firearm bullets, whereas 24.44% were due to traffic accidents. Ugboko *et al*. (36) reported that firearm bullets are the main reason for 2.7% of the fractures.

Some studies have also reported cranial injuries as the most common associated trauma (19,38,51). This relatively conforms with the current findings, which showed that cranial injuries were the second most prevalent type.

The prevalent method of treatment in our study was based on closed reduction (64.3%), which was relative to other results (14,15,51). No complications concerning occlusion and mouth opening were encountered in these patients.

For treatment of mandibular fracture, several methods of closed reduction were used for example the Ehrich’s arch bar, other interdental wirings and splints. In developing countries closed reduction are preferred by the people against the open reduction (52).

In the past 15 years, plate osteosynthesis has become popular in the management of facial fractures and in the treatment of mandibular fractures (53). Surgeons prefer it because it offers stable and precise anatomical reduction of fragments, allows immediate recovery of function as it has no IMF, shortens the period of bone healing and decreases the recovery period. Despite the obvious advantages, it has not become popular in many developing countries mainly because of cost factors. However, 35.7% of all maxillofacial fractures in our series were treated with open reduction and internal fixation. Patients treated with ORIF were routinely placed in inter maxillary fixation only intra-operatively. IMF was then released in all except for the cases which had concomitant condylar fractures, planned to treat conservatively with arch bars and IMF.

In our institute, open reduction and internal fixation using miniplates are the most preferred treatment plans for maxillofacial fractures. The technical and functional advantages of miniplate osteosynthesis over maxillomandibular fixation including the ease of use, precise anatomical reduction, limited or complete avoidance of maxillomandibular fixation, functional stability and improved mouth opening have made it more preferable (51).

Finally, the epidemiological study of facial trauma makes it possible to outline the risk situations, as well as the characteristics of individuals susceptible to this type of trauma. Moreover in the planning how to manage their patients, the evaluation of treatment effectiveness and the understanding of complications may provide a more realistic and consistent interpretation. It should be mentioned that besides to this fact that trauma should not only be seen exclusively as a medical condition, but also as a social and economic problem. Healthcare costs to treat victims, damage to property involved in the traumatic event, losses in wages, and permanent or transient disability often lead to difficulties in the reintegration and rehabilitation of victims into society and their return to work (1).

## Conclusions

Considering the geographic and cultural indices of the evaluated population, it can be concluded that the patients age and gender and trauma causes, significantly affect the prevalence of maxillofacial traumas and fracture types and so the best treatment plans. This would be useful for appropriate health care policy and management set up in every society.

## References

[1] Paes JV, de Sá Paes FL, Valiati R, de Oliveira MG, Pagnoncelli RM (2012). Retrospective study of prevalence of face fractures in southern Brazil. Indian J Dent Res.

[2] Rutland-Brown W, Langlois JA, Thomas KE, Xi YL (2006). Incidence of traumatic brain injury in the United States, 2003. J Head Trauma Rehabil.

[3] Simons-Morton B, Lerner N, Singer J (2005). The observed effects of teenage passengers on the risky driving behavior of teenage drivers. Accid Anal Prev.

[4] Aksoy E, Ünlü E, Sensöz Ö (2002). A retrospective study on epidemiology and treatment of maxillofacial fractures. J Craniofac Surg.

[5] Wittchen HU, Jacobi F, Rehm J, Gustavsson A, Svensson M, Jönsson B (2011). The size and burden of mental disorders and other disorders of the brain in Europe 2010. Eur Neuropsychopharmacol.

[6] Wood EB, Freer TJ (2001). Incidence and aetiology of facial injuries resulting from motor vehicle accidents in Queensland for a three-year period. Aust Dent J.

[7] Ribeiro M, Marcenes W, Croucher R, Sheiham A (2004). The prevalence and causes of maxillofacial fractures in patients attending Accident and Emergency Departments in Recife-Brazil. Int Dent J.

[8] Adebayo ET, Ajike OS, Adekeye EO (2003). Analysis of the pattern of maxillofacial fractures in Kaduna, Nigeria. Br J Oral Maxillofac Surg.

[9] Fasola AO, Nyako EA, Obiechina AE, Arotiba JT (2003). Trends in the characteristics of maxillofacial fractures in Nigeria. J Oral Maxillofac Surg.

[10] Al-Khateeb T, Abdullah FM (2007). Craniomaxillofacial injuries in the United Arab Emirates: a retrospective study. J Oral Maxillofac Surg.

[11] Erol B, Tanrikulu R, Görgün B (2014). Maxillofacial Fractures. Analysis of demographic distribution and treatment in 2901patients (25-year experience). J Craniomaxillofac Surg.

[12] Moncrieff NJ, Qureshi C, Deva AK (2004). A comparative cost analysis of maxillofacial trauma in Australia. J Craniofac Surg.

[13] Brasileiro BF, Passeri LA (2006). Epidemiological analysis of maxillofacial fractures in Brazil: A 5-year prospective study. Oral Surg Oral Med Oral Pathol Oral Radiol Endod.

[14] Motamedi MH (2003). An assessment of maxillofacial fractures: a 5-year study of 237 patients. J Oral Maxillofac Surg.

[15] Ansari MH (2004). Maxillofacial fractures in Hamedan province, Iran: a retrospective study (1987–2001). J Craniomaxillofac Surg.

[16] Kadkhodaie MH (2006). Three-year review of facial fractures at a teaching hospital in northern Iran. Br J Oral Maxillofac Surg.

[17] Mohajerani SH, Asghari S (2011). Pattern of mid-facial fractures in Tehran, Iran. Dent Traumatol.

[18] Zandi M, Khayati A, Lamei A, Zarei H (2011). Maxillofacial injuries in western Iran: a prospective study. Oral Maxillofac Surg.

[19] Mijiti A, Ling W, Tuerdi M, Maimaiti A, Tuerxun J, Tao YZ (2014). Epidemiological analysis of maxillofacial fractures treated at a university hospital, Xinjiang, China: a 5-year retrospective study. J Craniomaxillofac Surg.

[20] Leles JL, dos Santos EJ, Jorge FD, da Silva ET, Leles CR (2010). Risk factors for maxillofacial injuries in a Brazilian emergency hospital sample. J Appl Oral Sci.

[21] Gartshore L (2010). A brief account of the life of René Le Fort. Br J Oral Maxillofac Surg.

[22] Navabi N, Nakhaee N, Mirzadeh A (2010). Validation of a Persian Version of the Oral Health Impact Profile (OHIP-14). Iran J Public Health.

[23] Ashrafi Asgarabad A, Naghibzadeh Tahami A, Khanjani N (2012). Exposure to hand-held mobile phone use while driving among Iranian passenger car drivers: an observational study. J Inj Violence Res.

[24] Dimitroulis G, Eyre J (1991). A 7-year review of maxillofacial trauma in a central London hospital. Br Dent J.

[25] Oikarinen K, Ignatius E, Kauppi H, Silvennoinen U (1993). Mandibular fractures in northern Finland in the 1980s--a 10-year study. Br J Oral Maxillofac Surg.

[26] Lee MC, Chiu WT, Chang LT, Liu SC, Lin SH (1995). Craniofacial injuries in unhelmeted riders of motorbikes. Injury.

[27] Iida S, Kogo M, Sugiura T, Mima T, Matsuya T (2001). Retrospective analysis of 1502 patients with facial fractures. Int J Oral Maxillofac Surg.

[28] Subhashraj K, Ramkumar S, Ravindran C (2008). Pattern of mandibular fractures in Chennai, India. Br J Oral Maxillofac Surg.

[29] Lee K (2012). Global trends in maxillofacial fractures. Craniomaxillofac Trauma Reconstr.

[30] Cabalag MS, Wasiak J, Andrew NE, Tang J, Kirby JC, Morgan DJ (2014). Epidemiology and management of maxillofacial fractures in an Australian trauma centre. J Plast Reconstr Aesthet Surg.

[31] Bakardjiev A, Pechalova P (2007). Maxillofacial fractures in Southern Bulgaria–a retrospective study of 1706 cases. J Craniomaxillofac Surg.

[32] Taher AA (1993). Management and complications of middle and upper-third facial compound injuries: An Iranian experience. J Craniofac Surg.

[33] Haug RH, Adams JM, Conforti PJ, Likavec MJ (1994). Cranial fractures associated with facial fractures: a review of mechanism, type, and severity of injury. J Oral Maxillofac Surg.

[34] Tanaka N, Tomitsuka K, Shionoya K, Andou H, Kimijima Y, Tashiro T (1994). Aetiology of maxillofacial fracture. Br J Oral Maxillofac Surg.

[35] Cabrini Gabrielli MA, Real Gabrielli MF, Marcantonio E, Holuchi-Vieira E (2003). Fixation of mandibular fractures with 2.0 mm miniplates: Review of 191 cases. J Oral Maxillofac Surg.

[36] Ugboko VI, Odusanya SA, Fagade OO (1998). Maxillofacial fractures in a semi-urban Nigerian teaching hospital. A review of 442 cases. Int J Oral Maxillofac Surg.

[37] Gassner R, Tuli T, Hächl O, Rudisch A, Ulmer H (2003). Cranio-maxillofacial trauma: a 10 year review of 9,543 cases with 21,067 injuries. J Craniomaxillofac Surg.

[38] Arabion H, Tabrizi R, Aliabadi E, Gholami M, Zarei K (2014). A retrospective analysis of maxillofacial trauma in shiraz, iran: a 6-year- study of 768 patients (2004-2010). J Dent (Shiraz).

[39] Mesgarzadeh AH, Shahamfar M, Azar SF, Shahamfar J (2011). Analysis of the pattern of maxillofacial fractures in north western of Iran: A retrospective study. J Emerg Trauma Shock.

[40] Arangio P, Vellone V, Torre U, Calafati V, Capriotti M, Cascone P (2014). Maxillofacial fractures in the province of Latina, Lazio, Italy: Review of 400 injuries and 83 cases. J Craniomaxillofac Surg.

[41] Momeni H, Shahnaseri S, Hamzeheil Z (2011). Distribution assessment of maxillofacial fractures in trauma admitted patients in Yazd hospitals: An epidemiologic study. Dent Res J (Isfahan).

[42] van Hout WM, Van Cann EM, Abbink JH, Koole R (2013). An epidemiological study of maxillofacial fractures requiring surgical treatment at a tertiary trauma centre between 2005 and 2010. Br J Oral Maxillofac Surg.

[43] Huelke DF, Compton CP (1983). Facial injuries in automobile crashes. J Oral Maxillofac Surg.

[44] de Matos FP, Arnez MF, Sverzut CE, Trivellato AE (2010). A retrospective study of mandibular fracture in a 40-month period. Int J Oral Maxillofac Surg.

[45] Hussain K, Wijetunge DB, Grubnic S, Jackson IT (1994). A comprehensive analysis of craniofacial trauma. J Trauma.

[46] Lee JH, Cho BK, Park WJ (2010). A 4-year retrospective study of facial fractures on Jeju, Korea. J Craniomaxillofac Surg.

[47] Scherbaum Eidt JM, De Conto F, De Bortoli MM, Engelmann JL, Rocha FD (2013). Associated injuries in patients with maxillofacial trauma at the hospital são vicente de paulo, passo fundo, Brazil. J Oral Maxillofac Res.

[48] Joshi SR, Saluja H, Pendyala GS, Chaudhari S, Mahindra U, Kini Y (2013). Pattern and Prevalence of Maxillofacial Fractures in Rural Children of Central Maharashtra, India. A Retrospective Study. J Maxillofac Oral Surg.

[49] van As AB, van Loghem AJ, Biermans BF, Douglas TS, Wieselthaler N, Naidoo S (2010). Causes and distribution of facial fractures in a group of South African children and the value of computed tomography in their assessment. Int J Oral Maxillofac Surg.

[50] Lee KH, Steenberg LJ (2008). Equine-related facial fractures. Int J Oral Maxillofac Surg.

[51] Bali R, Sharma P, Jindal S, Sharma R (2010). Bone resorption after bioresorbable fixation of a fractured paediatric mandible - a case report. Oral Surg.

[52] Al Ahmed HE, Jaber MA, Abu Fanas SH, Karas M (2004). The pattern of maxillofacial fractures in Sharjah, United Arab Emirates: a review of 230 cases. Oral Surg Oral Med Oral Pathol Oral Radiol Endod.

[53] Bali R, Sharma P, Garg A, Dhillon G (2013). A comprehensive study on maxillofacial trauma conducted in Yamunanagar, India. J Inj Violence Res.

